# Development of artificial intelligence algorithms trained with real-world data for suicide risk prevention in pediatric and adult patients: The IDICIUS Project

**DOI:** 10.1192/j.eurpsy.2025.912

**Published:** 2025-08-26

**Authors:** C. Peña Gómez, M. Fradera, M. Caravaca, J.-F. Martínez, D. Roche, J. Giraldo, J. A. Escofet, E. Barberia, D. Palao

**Affiliations:** 1 Unitat de Neurociència Traslacional, Institut d’Investigació i Innovació Parc Taulí (I3PT-CERCA), Sabadell; 2 Institut de Neurociències (INc), Universitat Autònoma de Barcelona, Bellaterra; 3 Centro de Investigación Biomédica en Red de Salud Mental, Instituto de Salud Carlos III, Madrid; 4 Health Quality and Assessment Agency of Catalonia; 5 Research Institute for Evaluation and Public Policies (IRAPP), Universitat Internacional de Catalunya (UIC); 6 Institute of Legal Medicine and Forensic Sciences (IMLCFC), Barcelona, Spain

## Abstract

**Introduction:**

Suicide is currently the leading external cause of mortality in Europe and one of the main causes of premature death. Suicidal behavior is highly heterogeneous, making it difficult to predict in clinical practice. However, among people who die by suicide, 83% had attended primary care in the previous year, and 50% had done so within 30 days of their death. Moreover, well-known suicide risk factors are already recorded in routine electronic health records (EHRs). Therefore, the development and implementation of artificial intelligence tools can help improve the prevention of suicidal behavior.

**Objectives:**

The aim is to design a platform based on predictive artificial intelligence (AI) predictive models that allows preventing suicide risk, addressing both suicidal attempts and/or ideation, as well as deaths by suicide, using clinical, biological, and sociodemographic factors through real-world data (RWD) from EHRs in a large sample of pediatric and adult patients undergoing psychiatric treatment.

**Methods:**

This is a retrospective population-based study that uses structured and anonymized EHRs of a large sample of pediatric and adult patients who received mental health care between 2018 to 2024. The IDICIUS project consists of five work packages (Figure 1):

- Extraction and integration of data from different sources: EHRs from Mental Health and Primary Care services; database of outpatient medicines invoiced by pharmacies from the Catalan Health Service; and all suicidal behaviors from the Suicide Risk Code register and also suicide deaths.
- Predictive models creation and validation
- Integrated care platform design
- Design and plan feasibility
- Scientific dissemination of results

**Results:**

We have identified approximately 40,000 patients who visited the Mental Health service at Parc Taulí Hospital between 2018 and 2024, of whom 162 died by suicide. The integration of these databases (Figure 2) has been achieved through the collaboration and coordination of three different public entities over the course of more than a year, ensuring compliance with legal and ethical standards at each step, given the sensitive nature of the data and its intended use in the development of an AI predictive algorithm.

**Image 1:**

**Figure:**
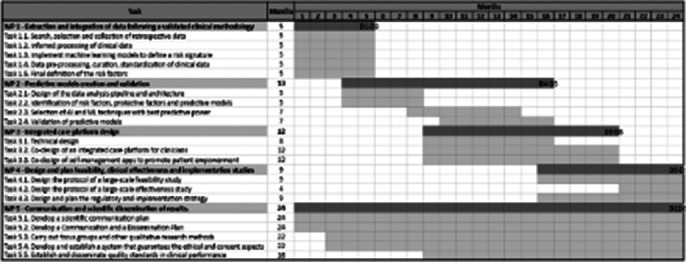

**Image 2:**

**Figure:**
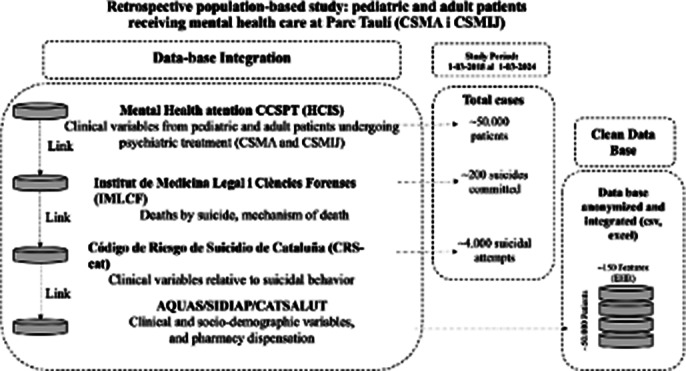

**Conclusions:**

We are now moving forward with the subsequent phases, aiming to make a decisive contribution to the more effective and efficient management of suicidal behavior in clinical populations. The potential clinical utility of the results obtained in the prediction of suicide is analyzed, applying the AI algorithm in real clinical practice conditions. The aim is to improve the information available, through the use of alarms that help clinicians in decision-making to improve suicide prevention in primary care (risk detection) and in specialized mental health care (detection of significant variations in baseline risk).

**Disclosure of Interest:**

None Declared

